# Green synthesis of silver nanoparticles from Kedrostis nana (Lam.) Cogn. extracts and its biological activities

**DOI:** 10.3389/fchem.2026.1894101

**Published:** 2026-08-20

**Authors:** Kareshma Doolabh, Yougasphree Naidoo, Nirasha Nundkumar, Karen Pillay

**Affiliations:** School of Agriculture and Science, University of KwaZulu-Natal, Durban, South Africa

**Keywords:** antibacterial, anticancer, antioxidant, cytotoxicity, green synthesis, silver nanoparticles

## Abstract

The medicinal plant *Kedrostis nana* is a relatively understudied species that belongs to the Cucurbitaceae family. It is important to understand the pharmacological potential of traditional medicinal plants such as *K. nana* as it could have potential in various clinical applications, especially since there have been so many advancements in the biomedical sector. Nanoparticles have also become of interest due to their size and various applications as antimicrobial agents, anticancer agents, and drug delivery agents. This study evaluated the biosynthesis of silver nanoparticles (AgNPs) from *K. nana* aqueous leaf, stem and tuber extracts. Characterization was accomplished using ultra-violet visible spectrometry, energy-dispersive x-ray spectroscopy (EDX), Fourier transform infrared spectroscopy (FTIR), high resolution transmission electron microscopy (HRTEM), dynamic light scattering (DLS) and zeta potential analysis. The synthesized AgNPs were explored for their antioxidant, antibacterial and cytotoxic potential. The results showed that the AgNPs were stable, negatively charged and polydispersed with sizes ranging from 21 to 28 nm. FTIR results revealed various functional groups present which could be attributed to the capping of the AgNPs by the plant’s phytochemicals. The stem and tuber AgNPs displayed significant antioxidant potential at 200 μg/mL which were similar to the known antioxidant, ascorbic acid. When tested against five bacterial strains, the tuber AgNPs appeared to outperform all other biosynthesized AgNPs as well as the positive control, neomycin. The cytotoxicity investigation exhibited the possibility of the tuber AgNPs being used as an anticancer agent. At 200 μg/mL, the AgNPs derived from the tuber displayed toxicity to the HeLa cell line. The tuber AgNPs also exhibited toxicity to the non-cancerous cell line, Hek293, however, further investigations are necessary such as apoptosis detection and gene expression profiling. These results corroborate the benefit of this plant’s use in traditional medicine.

## 1 Introduction

In the field of science and engineering, nanotechnology provides a sustainable solution for many challenges posed in various sectors such as medicine, agriculture, catalysis and industrial activities (Mata et al., 2012; Beyene et al., 2017). However, the field of nanotechnology is evolving, and researchers are actively developing, synthesizing and manipulating various materials at a nano level (Fahim et al., 2024). Nanoparticles (NPs) fall between the microscopic and mesoscopic level, and it is widely agreed that they range in size from 1 to 100 nm (Abbasi et al., 2016; Beyene et al., 2017). NPs are used in various fields such as biomedicines, pharmaceuticals, catalysis, cosmetics, food technology, sensors and electrochemistry (Vanlalveni et al., 2021). Metal noble element NPs synthesized from silver, gold and palladium have become a promising trend in nanotechnology, especially for creating biomaterials used in cutting-edge diagnostic tools and devices for the treatment of various severe diseases (Burlec et al., 2023). Metal NPs, especially silver NPs (AgNPs), have increased in popularity due to their exclusive properties, such as the size and shape which affects their physical and chemical properties, ability to absorb and scatter light in the near-infrared and visible light range, magnetism, optical and electrical properties (Altammar, 2023).

AgNPs have been incorporated into antimicrobial applications where they are used in antiseptic sprays, bandages and catheters, they are also used as agents in anticancer treatment, cosmetic products, biosensor materials, food storage, composite fibers and electronic components (Wei et al., 2015; Abbasi et al., 2016; Shayo et al., 2024). Physical and chemical synthesis methods are efficient in creating well-defined AgNPs. Physical methods include ball milling, laser ablation, ultrasonication, lithography, evaporation-condensation, and arc discharge, which provide various sizes and shapes of AgNPs. Chemical synthesis includes methods such as chemical reduction, co-precipitation, solvothermal, electrochemical reduction, chemical vapor deposition and micelles, which reduces Ag^+^ to Ag atoms with no charge (Ealias and Saravanakumar, 2017). However, these methods do have some major drawbacks such as long synthesis time, difficulty in purification, increased production cost, and the discharge of dangerous and biohazardous by-products. Additionally, chemical synthesis can also have negative consequences in medical applications such as hazardous chemical species that could be absorbed during the synthetic process (Parashar et al., 2009; Lee and Nagajyothi, 2011; Kumari and Sarkar, 2021).

In order to overcome these limitations, simple, cost effective, environmentally friendly and reliable biological alternatives have emerged. Moreover, the high yield production of size-defined AgNPs using biological systems has gained much attention (Burlec et al., 2023). Green synthesis of AgNPs utilizes plants and microorganisms (bacteria, fungi and yeast). In this green method, the leaves, roots, stems, bark, fruit and flowers are mechanically ground into a powder and extracted using solvents such as water (Akhtar et al., 2013). The process yields a solution containing bioactive compounds such as alkaloids, phenols, and terpenoids, which are used as a stabilizing agent for AgNP synthesis (Fahim et al., 2024). This approach supports sustainability as it employs renewable resources, conserves energy, has low environmental impact, and works under ambient conditions.

The flora of South Africa is vibrant due to the vast differences in climatic and environmental conditions and many of these indigenous plants have promising therapeutic potential (Osunsanmi et al., 2022; Gaobotse et al., 2023). The indigenous plant *K. nana* is a plant that is yet to be explored. This deciduous plant is endemic to low altitudes alongside the coastal regions of KwaZulu-Natal to the Western Cape in South Africa (Manning and Goldblatt, 1998). According to World Flora Online (2024), *K. nana* is a perennial with fleshy, suborbicular-cordate-shaped leaves. The plant is a climber supported by a tuber and annual stems with tendrils (Deutschlander et al., 2009). Three intraspecies have been officially accepted (Germishuizen and Meyer, 2003). According to literature, *K. nana* has been utilized in traditional systems to aid in the treatment of hypertension, constipation, malaria, ulcers, menstrual complaints, syphilis, diabetes, cancer, hepatitis, and also as a diuretic (Watt and Breyer-Brandwijk, 1932; Hutchings et al., 1996; van Wyk and Gericke, 2000; van Wyk, 2008; Deutschlander et al., 2009; van Wyk et al., 2009; Philander, 2011; Omokhua-Uyi and Van Staden, 2020). None of the medicinal properties of *K. nana* have been scientifically investigated to validate the plants’ pharmacological capabilities. Although there are several species within the Cucurbitaceae family, including *Citrullus colocynthis*, *Lagenaria siceraria* and *Momordica charantia*, have been investigated for their ability to synthesize AgNPs. There is a dearth of information regarding AgNPs using *K. nana*. To the best of our knowledge, this would be the first study to investigate the synthesis of AgNPs using the leaf, stem and tuber aqueous extracts as well as to evaluate their physiochemical characteristics coupled with their antioxidant, antibacterial and cytotoxic properties. This study expands the purpose of *K. nana* in green technology; it also provides insight into the influence of different plant organs on NP synthesis and biological activity.

## 2 Materials and methodology

### 2.1 Materials

*K. nana* leaves, stems, and tubers were collected by Dr Adriaan Grobler and Ms. Nicole Zee Loebenberg. Dr Grobler collected material from the coastal dune cordon between Kini Bay and Lauries Bay (−34.024960°, 25.387570°) and Baakens River Valley (−33.968092°, 25.585866°) in Qqeberha. Ms. Nicole Zee Loebenberg collected material in an area located close to the !Khwa ttu San Heritage Centre (−33.354952°, 18.270046°) in Yzerfontein, Western Cape. All the collected plant materials were identified against herbarium specimens. For later use, two to three live plants were propagated in a garden in Newlands West, KwaZulu-Natal. A voucher specimen (UDW23002) was transferred to the Ward Herbarium in the School of Agriculture and Science at the University of KwaZulu-Natal.

The solvents, reagents, and media used were of analytical grade and procured from Sigma-Aldrich (Germany) unless otherwise stated. The following strains were from the American Type Culture Collection (ATCC): *Enterococcus faecalis* (ATCC 5129), *Escherichia coli* (ATCC 35218), *Staphylococcus aureus* (ATCC 43300) and *Pseudomonas aeruginosa* (ATCC 27853).

### 2.2 Extract preparation

The plant material was separated according to leaves, stems and tubers. The material was left to dry in an oven for 3 days at 30 °C. Once dry, it was placed separately into a blender (600 W glass jug Russell Hobbs 23821-56 blender, Russell Hobbs Inc., United Kingdom) and ground into a fine powder. The various plant powders were used for aqueous extraction.

For the extraction process, 10 g of ground plant material was deposited into a conical flask, and 100 mL of distilled water was poured over the plant material. The flask was covered entirely in foil and placed onto a mechanical shaker at a speed of 110 rpm, with temperature set at 30 °C-32 °C and left for 24 h. The crude extract was filtered using the Whatman® No. 1 filter paper. The procedure was repeated twice and extraction from each plant organ was carried out separately. Approximately 20 mL of each extract was placed into an oven to dry for 3 days at 50 °C. The resultant dried extract was suspended in sterile distilled water and used for further testing.

### 2.3 Biosynthesis of nanoparticles

AgNPs biosynthesis was carried out using the procedures described by Amutha et al. (2014) and Premasudha et al. (2015), with some modifications. Briefly, 100 mL of 10 mM silver nitrate (AgNO_3_) was made up with distilled water, dispensed into a conical flask and mixed with the *K. nana* aqueous extract in a 1:1 ratio. The subsequent solution was placed into a water bath set at 80 °C for 1 h. The mixture was centrifuged for 15 min at 4,000 rpm, and the supernatant was discarded. The resultant pellet was topped with 0.5 mL sterile distilled water and left to dry for 48 h at 50 °C. This dried pellet was dissolved in 10% DMSO and was used for characterization and subsequent analyses. The process was carried out separately for the aqueous leaves, stems, and tuber extracts.

### 2.4 Characterization of silver nanoparticles

#### 2.4.1 UV- spectrometry and color change

UV-spectrometry of the AgNPs was carried out at the start of synthesis prior to placing the flask into the water bath, 30 min into the synthesis process and after 1 h (end of the process). The wavelength range of 320-620 nm was used, and blanks comprised of the plant aqueous extract and AgNO_3_ solution (10 mM). A change in solution color to brown was also an indication of successful synthesis of the nanoparticles. This method was carried out as described by Verma and Mehata (2016) and Zabihi et al. (2024), with some modifications.

#### 2.4.2 Scanning electron microscopy and energy-dispersive X-ray spectroscopy (EDX)

One drop of the synthesized AgNPs was pipetted onto aluminium stubs to dry. Subsequently, gold was used to coat the stubs using the Polaron SC500 Sputter Coater (Quorum Technologies Ltd., United Kingdom). The elemental composition of the AgNPs was identified using the EDX detector Zeiss Plus Field Emission Gun Scanning Electron Microscope (FEG-SEM) (Carl Zeiss, Germany). This method was carried out as described by Premasudha et al. (2015), with some modifications.

#### 2.4.3 High-resolution transmission electron microscopy (HRTEM)

The morphology of the AgNPs was analyzed using methods adapted from (Ahmad et al., 2016). The synthesized AgNPs were sonicated for 10 min. Carbon coated copper grids were immersed into the AgNP solutions and placed under a lamp for solvent evaporation for 15 min. The grids were viewed using a JEOL High-Resolution Transmission Electron Microscope (HRTEM) 2100 (JEOL Ltd., United States) at 200 KeV. The sizes of the NPs were measured using ImageJ software.

#### 2.4.4 Dynamic light scattering (DLS) and zeta potential analysis

The size and stability of the AgNPs were determined using the DLS and zeta potential techniques on the Malvern Zetasizer Software version 8 (England) as adapted from Premasudha et al. (2015).

#### 2.4.5 Fourier transform infrared spectroscopy (FTIR)

The functional groups present in the biosynthesized AgNPs solution of the plant part were assessed using the Perkin Elmer Spectrum 100 spectrophotometer (Perkin Elmer Inc., United States) as reported by Iyer and Panda (2018).

### 2.5 Antioxidant activity via DPPH analysis

The synthesized AgNPs of each plant organ (leaves, stems and tubers) were prepared separately using 10% DMSO for the initial stock. The stock extract (100 µL) was pipetted into a 96-well microplate, followed by serial dilution to attain concentrations of 6.25–200 μg/mL. The DPPH solution (0.2 mM of 2, 2՛-diphenyl-1-picrylhydrazyl) was prepared and thereafter pipetted (50 µL) into the wells of the microplate. The extract and DPPH solution were mixed thoroughly, and the microplates were covered in foil and placed in the dark at 23 °C for 30 min. Subsequently, the microplates were analyzed at a wavelength of 520 nm. This procedure was completed for the AgNPs synthesized from each plant part. The biosynthesized AgNPs at each concentration was used as the experimental blank, and ascorbic acid was used as the positive control. Three biological replicates were performed for reproducibility, and 5 technical replicates were conducted for each sample. To calculate the percentage scavenging activity, the following equation was used:

$$\%\text{ Scavenging activity}=\frac{\text{Absorb}.\text{control}-\text{Absorb}.\;\text{sample}}{\text{Absorb}.\;\text{control}}\;x\;100$$

### 2.6 Antibacterial activity

#### 2.6.1 Bacterial strain preparation

The bacterial strains were prepared using methods proposed by Samie et al. (2005) with some modifications. The bacterial strains were initially grown in liquid culture of Mueller Hinton (MH) broth (Rochelle, Johannesburg, South Africa) in an incubator set at 31 °C for 18 h. An inoculum (10%) was prepared using 5 mL of the grown bacteria and 45 mL of MH broth. The mixture was vortexed and incubated at 31 °C for 18 h. Thereafter, the grown bacterial inoculum was centrifuged at 4,000 rpm for 15 min with the temperature set at 4 °C. The supernatant was discarded. The remaining bacterial pellet was resuspended in MH broth and adjusted to a 0.5 McFarland standard with an optical density of 0.08–0.1, corresponding to approximately 1–2x 10^8^ CFU/mL. The bacterial strains used included the Gram-negative *E. coli*, *Klebsiella pneumonia* and *P. aeruginosa* strains, and Gram-positive *E. faecalis* and *S. aureus* strains.

#### 2.6.2 INT assay

The INT assay method was adapted from Samie et al. (2005). A stock solution of biosynthesized AgNPs was prepared for each plant part in 10% DMSO and serially diluted in the microplate to attain concentration ranges from 6.25 to 200 μg/mL. Following dilution, the final concentrations of DMSO in the wells ranging from 0.0625% to 2%. The bacterial cultures (80 µL) were added to each well of the microplate, followed by incubation at 31 °C for 18 h. The total well volume was 100 µL before incubation. After incubation, 40 µL of 0.2 mg/mL 2-(4-iodophenyl)-3-(4-nitrophenyl)-5-phenyl-2H-tetrazolium) or p-Iodonitrotetrazolium (INT) was added to each well and mixed thoroughly. The microplates were incubated at 31 °C for 30 min. Thereafter, 100 µL of 5% sodium dodecyl sulphate (SDS) solution was pipetted into the microplates, mixed and further incubated for 3 h at 31 °C. The AgNP-INT-SDS mixture was read at a wavelength of 490 nm. The procedure was conducted for each plant organ biosynthesized AgNPs and each bacterial strain. Five technical and three biological replicates were performed. The experimental blanks used were the serially diluted biosynthesized AgNPs in MH broth without bacteria. These corrected any background absorbance contributed by the AgNPs. The positive control/standard was neomycin at the same concentrations. Neomycin was selected due to it is a broad-spectrum aminoglycoside that exhibits antibacterial activity against Gram-positive and -negative bacterial strains. The growth control consisted of the bacterial suspension in MH broth without AgNPs. The percentage toxicity was calculated as follows:

$$\%\text{ Toxicity}=100-\left(\frac{\text{Absorb}.\;\text{sample}-\text{Mean}\;\text{absorb}.\;\text{blank}}{\text{Mean}\;\text{absorb}.\text{negative}\;\text{control}}\;\times 100\right)$$

The minimum inhibitory concentration was determined by comparing the mean optical density (OD) values to a calculated MIC threshold. If the mean OD value at a given concentration was below the threshold, then that concentration corresponded to the MIC. The MIC threshold was calculated as follows:

$$\text{MIC Threshold}=\text{Mean optical denisty control}+\left(2\;\times \text{ Standard deviation}\right)$$

### 2.7 Cytotoxicity assay

The cytotoxicity assay was implemented using a modified protocol described by Naidoo et al. (2022). The cell lines used were Hek293 and HeLa. These cells were maintained in Eagle’s Minimum Essential Medium (EMEM) combined with 10% (v/v) gamma-irradiated Fetal Bovine Serum (FBS) and 1% antibiotics (100 U/mL penicillin and 100 μg/mL streptomycin). The cells were incubated at 37 °C with 5% CO_2_ in a HEPA Class 100 Steri-Cult CO_2_ incubator (Thermo-Electron Corporation*,* Waltham, Massachusetts, United States). For the MTT (3- [4.5-dimethylthiazol-2-yl]-2.5 diphenyl tetrazolium bromide) assay, the cells were dissociated using trypsin and seeded into 96-well sterile plates. The seeding density was approximately 3 × 10^4^ cells/well. The seeded plates were incubated to allow for attachment for 24 h at 37 °C. Fresh medium was dispensed to replace the old medium, and the respective serially diluted aqueous extract synthesized AgNPs (6.25–200 μg/mL) were dispensed into the well. The plates were left to incubate at 37 °C for 48 h. The negative controls were set up with one containing only cells (100% cell survival/viability) and the other containing 10 µL of 10% DMSO. This resulted in a final concentration of 0.5% DMSO per well.

After incubation, the spent medium was discarded. Fresh medium (100 μL) was dispensed into the wells to replace the spent medium, followed by the addition of 10 μL MTT reagent (5 mg/mL in phosphate-buffered saline) into each well. The plates were incubated for a further 4 h at 37 °C. Subsequently, the MTT medium solution was removed and 100 μL DMSO was added to solubilize the formazan crystals. The absorbance was read at 540 nm utilizing a Mindray MR-96 A microplate reader (Vacutec, Hamburg, Germany). The assay was also carried out on the leaves, stems and tuber AgNP solutions. Percentage cell viability versus AgNP concentrations were calculated using Microsoft Excel. These calculations are provided in the supplementary material.

### 2.8 Statistical analyses

The biological assays were performed in triplicate for each of the plant organ biosynthesized AgNP solution. The means were calculated using GraphPad Instat. A two-way Analysis of Variance (ANOVA) with Tukey’s post-hoc test was conducted using GraphPad Prism Version 10 to verify significant differences within the samples and concentrations. The value p < 0.05 was considered statistically significant and the asterisk indicated the level of significance (*- significant, **- very significant, ***- highly significant, **** extremely significant). The IC_50_ values for the cytotoxicity assay were determined using the percentage toxicity calculated and a linear regression model on Microsoft Excel. The concentration that corresponded to 50% cell viability was calculated using the equation of the fitted linear trendline and provided in the supplementary material. For AgNPs that did not produce 50% inhibition within the concentration range, the calculated IC_50_ values denoted extrapolated approximations and were interpreted as exceeding the highest concentration tested.

## 3 Results and discussion

### 3.1 Characterization of biosynthesized nanoparticles

#### 3.1.1 Ultraviolet spectroscopy and color change

**FIGURE 1:**
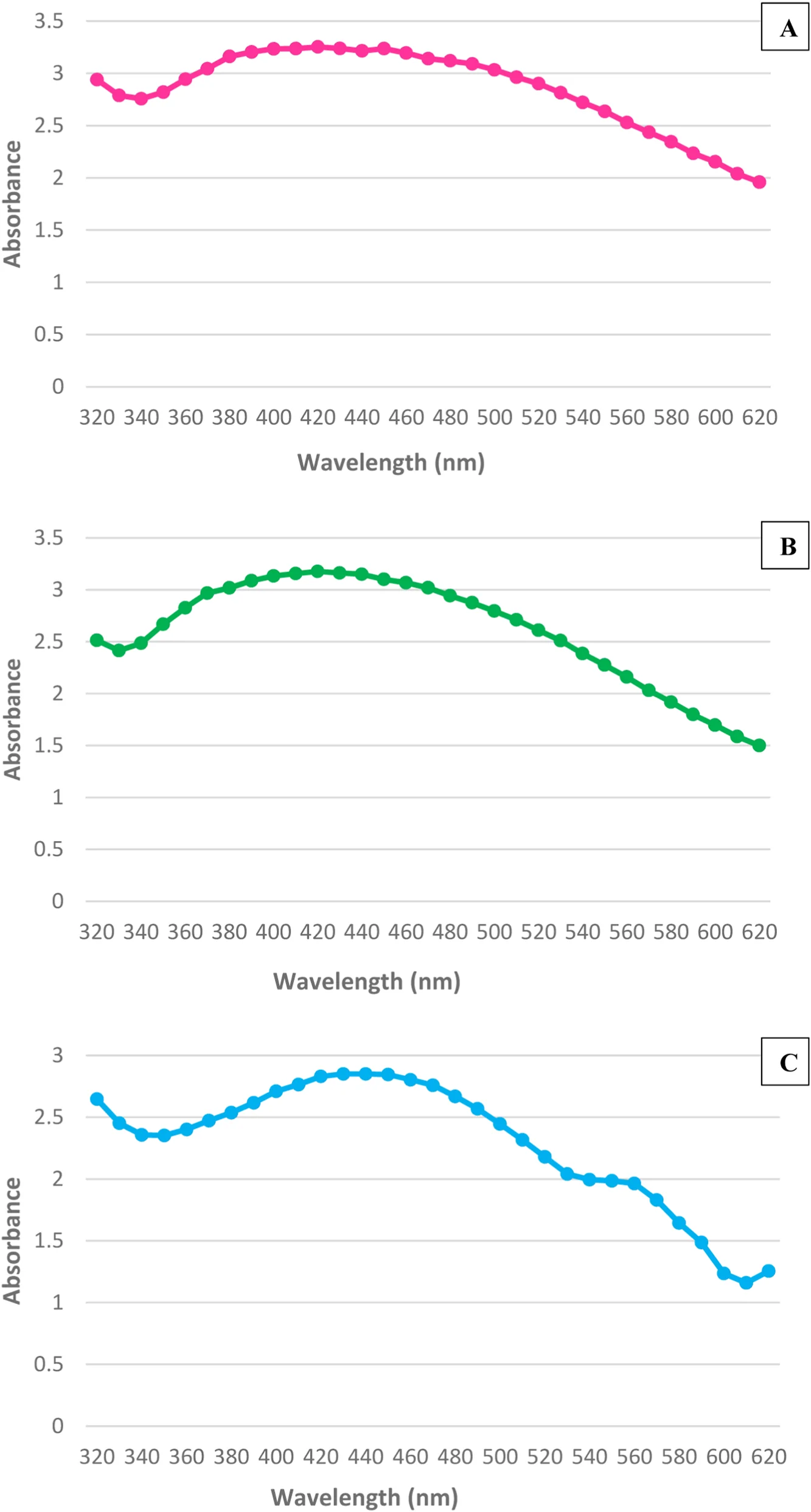
UV spectroscopy of silver nanoparticles synthesized from *Kedrostis nana* leaves **(A)**, stems **(B)** and tuber **(C)** extract.

**FIGURE 2:**
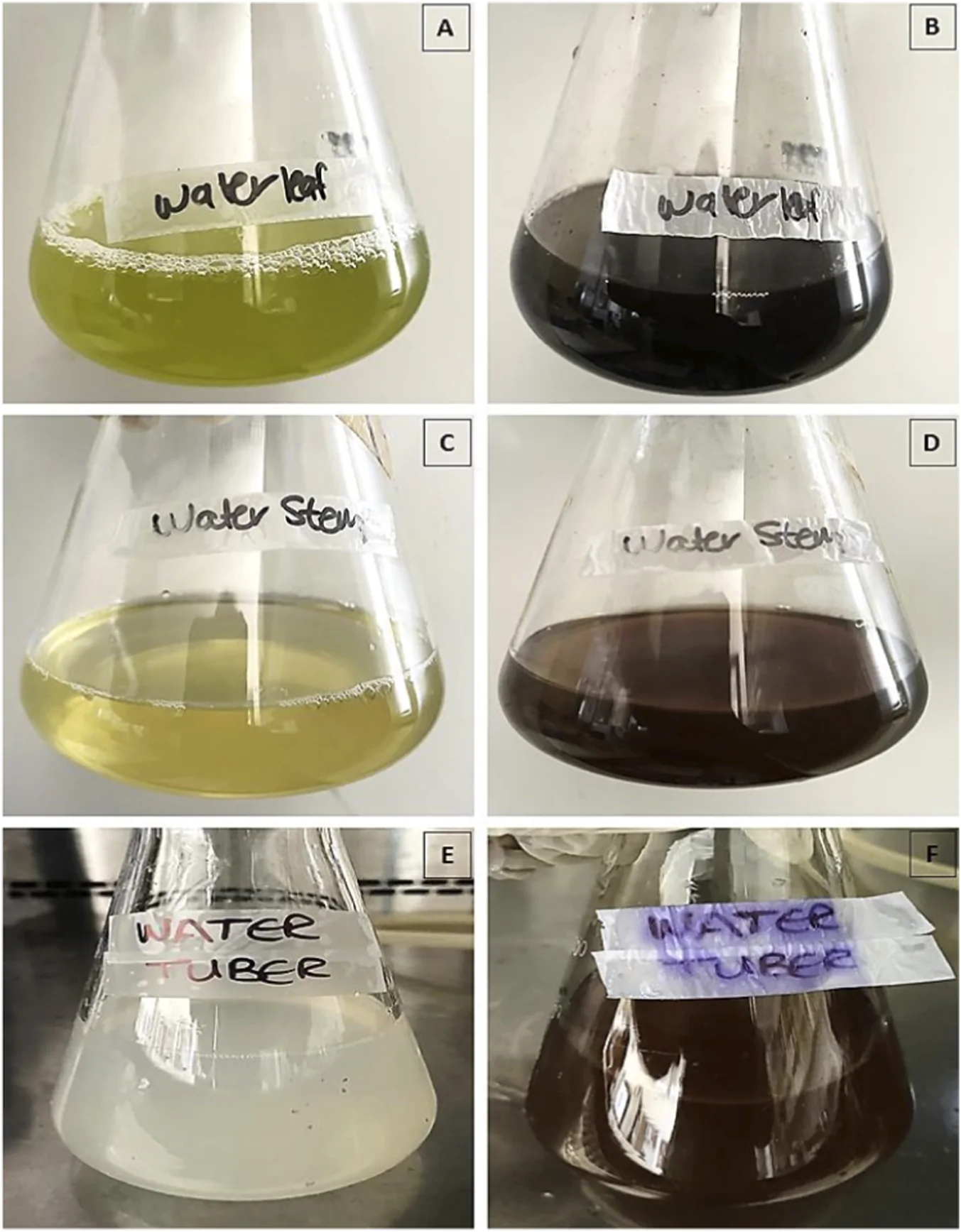
Color change of silver nanoparticles synthesized from *Kedrostis nana* aqueous extracts before and after boiling for 1 h **(A,B)** Leaf extract sample before and after synthesis respectively; **(C,D)** Stem extract sample before and after synthesis respectively; **(E,F)** Tuber extract sample before and after synthesis respectively.

In the present study, *K. nana* plant aqueous extracts were used to biosynthesize AgNPs. UV-visible spectroscopy was one method used to characterize the NPs synthesized. The surface plasmon resonance was measured between 320-620 nm to investigate AgNP formation. From the spectral analysis, it was observed in Figures 1A,B and C that peaks formed between 340 nm and 530 nm for the leaf, stem and tuber extracts. This indicates a broad band absorption of the AgNPs. The highest peaks are observed at 420 nm for the leaf and stem AgNPs and at 430 nm for the tuber AgNPs. In Figure 2, it can be observed that the initial colors of the plant extract and AgNO_3_ solution at 0 min of synthesis of AgNPs were green, yellow-brown and white for the leaf, stem and tuber, respectively. After an hour in the water bath, all three solutions had changed to a deep brown color.

According to literature, it is typical for AgNPs to reach the highest capacity of absorption between 420-450 nm. This is indicative of AgNP formation (Al-Otibi et al., 2021). The broad absorption band observed may be attributed to the dispersion of the particle shape and/or size rather than a uniform population. Additionally, the broadened band may also be due to NP aggregation as well as the adsorption of the phytochemicals from the extracts onto the surface of the NP. This factor has influences on the local dielectric environment and plasmon coupling (Kelly et al., 2003; Lee and El-Sayed, 2006; Ssekatawa et al., 2021). These results fall within the expected absorption range hence it can be concluded that AgNPs were biosynthesized from *K. nana* extracts. From previous studies it has been observed that NPs formed in the 420-450 nm surface plasmon resonance wavelength range comprise of sizes between 2-100 nm (Iqbal et al., 2020; Palithya et al., 2022).

Colour changes observed could be explained by two reasons, firstly, the plant extract acted as a capping and stabilizing agent which reduced Ag^+^ to Ag^0^ ions (Ahmad et al., 2016). Secondly, the excitation of the surface plasmon resonance effect attribute to the color change (Liaqat et al., 2022; Shereen et al., 2024). The surface electrons collectively oscillate at a certain frequency causing the NPs to absorb and scatter light resulting in the reflected light appearing brown (Kelly et al., 2003; Evanoff and Chumanov, 2005; Jain et al., 2006; Hang et al., 2024). Some factors that control the color emitted are: (A) the particles size where smaller particles appear yellow while larger particles look brown, (B) the aggregation often deepens the color of the solution, (C) the particle plays a role in absorbing different wavelengths, and (D) the capping solution such as plant extracts tend to alter the local refractive index which results in a shift in the surface plasmon resonance band (Kaur et al., 2020; Sharma et al., 2020; Sørensen et al., 2022; Car and Krstulović, 2022; Alzoubi et al., 2023).

#### 3.1.2 Transmission electron microscopy and EDX analysis of biosynthesized silver nanoparticles

**FIGURE 3:**
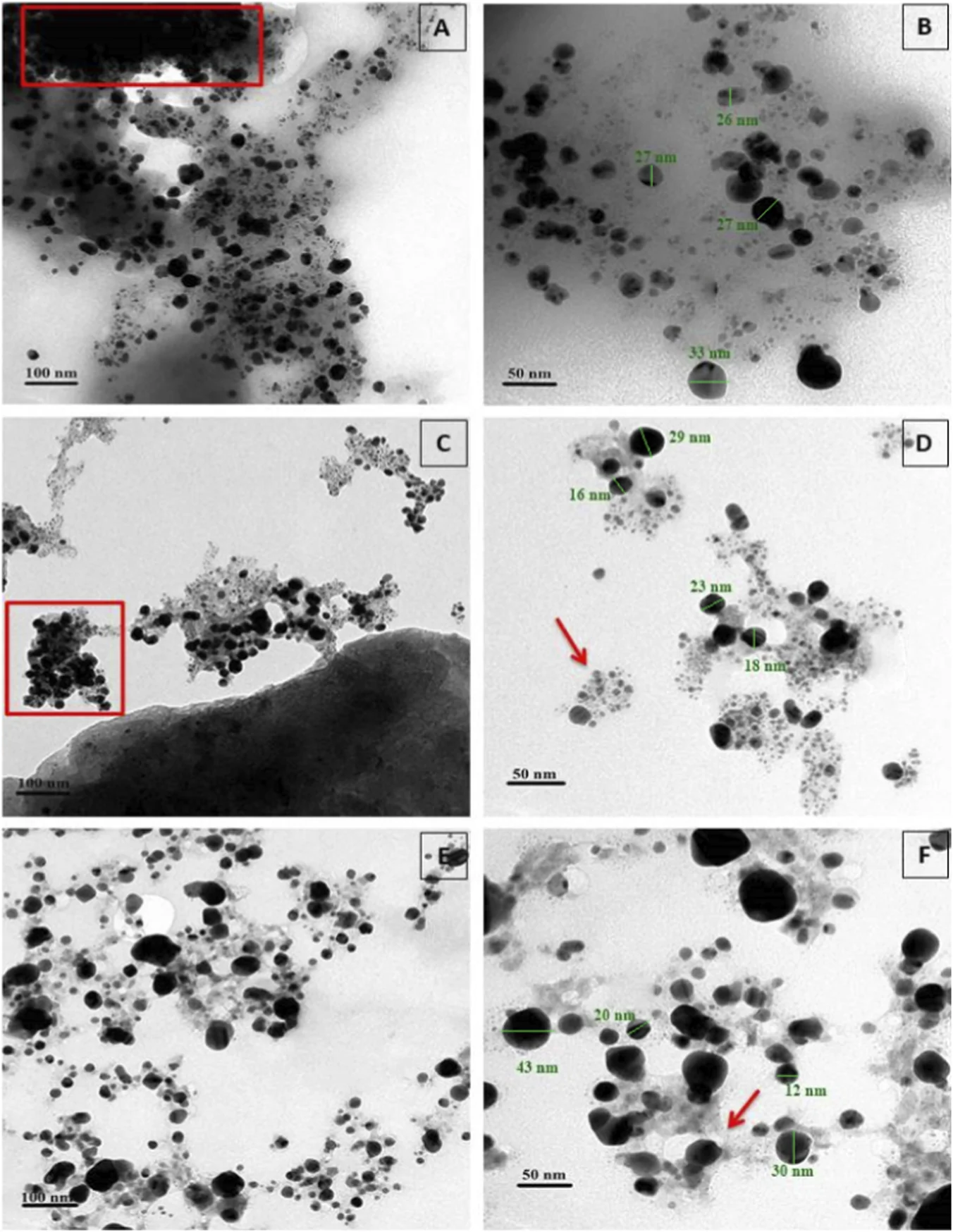
Transmission electron micrographs of synthesized silver nanoparticles from *Kedrostis nana* extracts. **(A,B)** AgNPs derived from leaf extract; **(C,D)** AgNPs derived from stem extract; **(E,F)** AgNPs derived from tuber extract. Red square indicating agglomeration; red arrow displaying biofilm.

**FIGURE 4:**
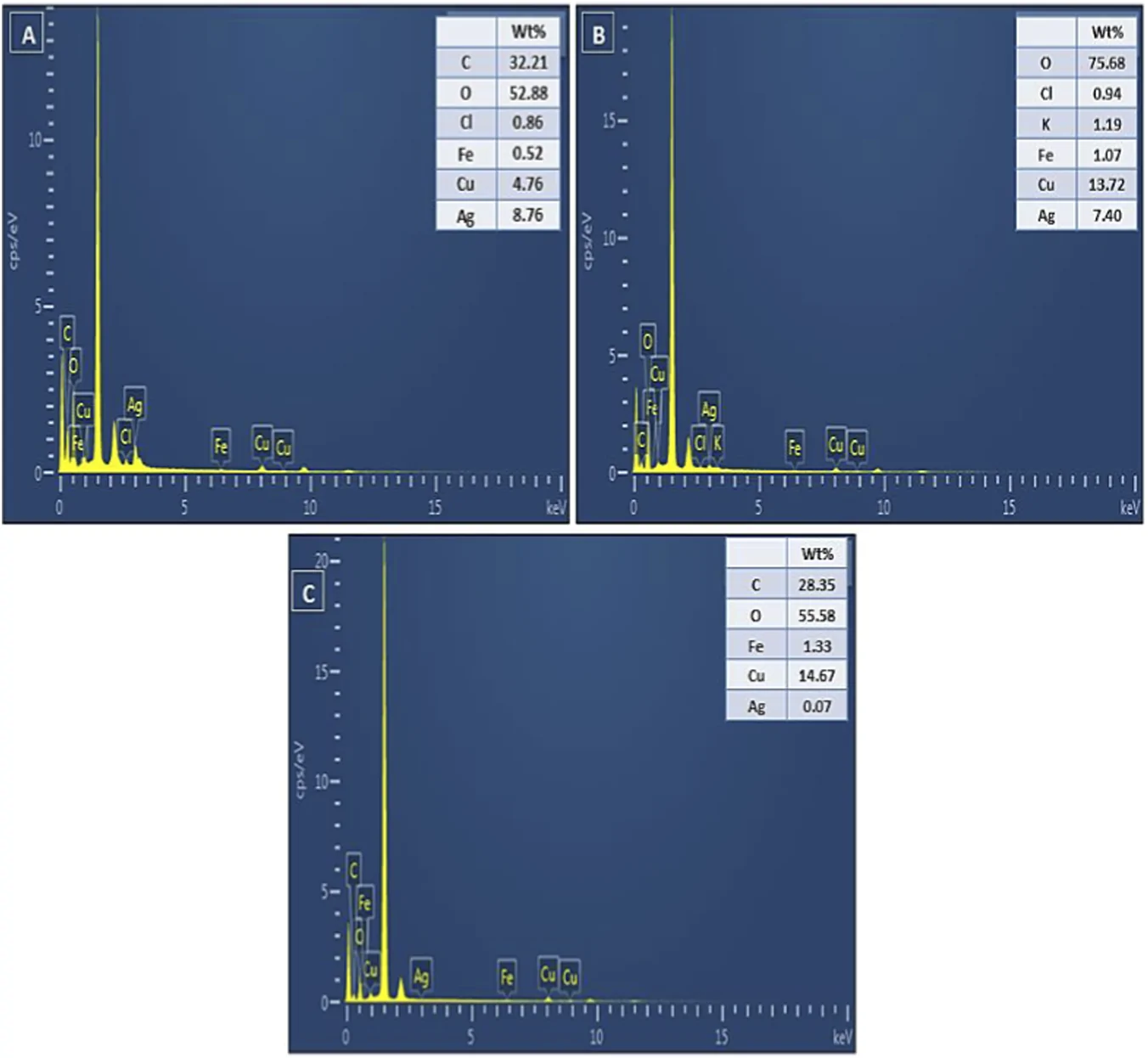
Elemental composition of silver nanoparticles synthesized by *K. nana* extracts. **(A)** AgNPs derived from leaf extract; **(B)** AgNPs derived from stem extract; **(C)** AgNPs derived from tuber extract.

The morphology of the AgNPs is presented in Figure 3. It is clearly demonstrated that the shape of these NPs is somewhat spherical. The size of the AgNPs vary among the different plant organs used to biosynthesize the NPs. There is agglomeration of the AgNPs demonstrated by the red squares in Figures 3A,B. Based on the NPs measured in the image (Figure 3B), the average size of the AgNPs biosynthesized from the leaf aqueous extract was 28 nm. In Figure 3D, the average size of the AgNPs biosynthesized from the stem extract was 21 nm. The average size of the AgNPs biosynthesized from the tuber extract was 26 nm (Figure 3F). The elemental composition of the AgNPs biosynthesized from *K. nana* extracts was determined via EDX analysis and can be observed in Figure 4. The Ag atoms present in the AgNPs showed peaks at 3 keV (Figures 4A–C). This is consistent with literature which indicates that AgNPs display prominent optical peaks at 3–4 keV (Kavaz et al., 2018; Siddique et al., 2024). The EDX analyses also revealed that the AgNPs in the leaf, stem and tuber (Figures 4A–C), which corresponded to the TEM Figures 3B,D,F, did not show peaks for nitrogen which is indicative of trace ions from AgNO_3_ being absent in the AgNP sample (Urnukhsaikhan et al., 2021). Other peaks were present for elements such as carbon, copper and iron. This is not unusual due to the presence of carbon coated copper grids (Singh et al., 2020). The EDX spectra exhibit a high oxygen content; this does not, however, indicate the formation of silver oxide NPs. The detected oxygen may be attributed to the biomolecules of the plant extracts which contain oxygen and have adsorbed onto the nanoparticle surface, the carbon coated grids, residual organic matter, absorbed water or oxygen surface species. Alternatively, this could also be due to the development of a thin oxide layer when the AgNPs were exposed to air (Naveed et al., 2024).

The biosynthesis of the AgNPs was carried out in an 80 °C water bath. Synthesis conducted in temperatures between 70 °C to 90 °C cause the formation of many nucleation centers which speeds up the reaction process. This allows for maximum production yield and more stable, smaller NPs (Elemike et al., 2017; 2019; Liu et al., 2020; Phuong et al., 2020; Waiezi et al., 2023; Stozhko et al., 2024). The agglomerations could be due to a lack of agitation of the sample before imaging, some abnormalities during the capping process of NP formation or sedimentation during the last stages of synthesis (Ahmad et al., 2013; Lateef et al., 2016; Bhole et al., 2023; Stozhko et al., 2024; Bibi et al., 2025).

#### 3.1.3 Dynamic light scattering and zeta potential analysis

**FIGURE 5:**
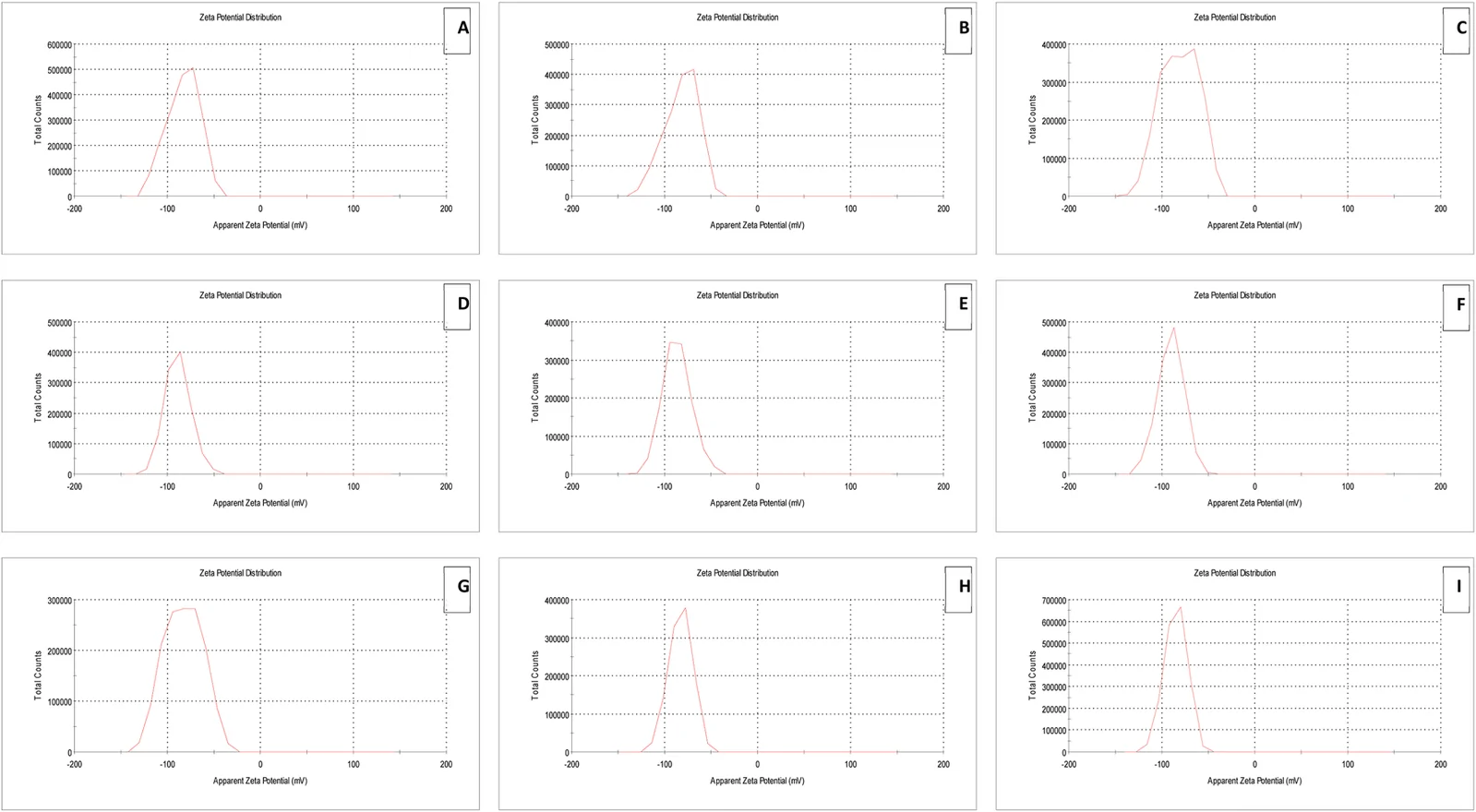
Zeta potential silver nanoparticles synthesized by *K. nana* extracts. **(A–C)** AgNPs derived from leaf extract; **(D–F)** AgNPs derived from stem extract; **(G–I)** AgNPs derived from tuber extract.

The zeta potential measurements of the leaf, stem and tuber-derived AgNPs are exhibited in Figures 5A–I. There are three independent measurements for each plant organ, i.e., leaf-AgNPs in Figures 5A–C, stem-AgNPs in Figures 5D–F and tuber-AgNPs in Figures 5G–I. All zeta potential replicates demonstrate good reproducibility of the green synthesized AgNPs. According to the results in Table 1, the mean zeta potential showed high negative values of −81.08, −88.5, and −83.5 for the leaf, stem and tuber biosynthesized AgNPs, respectively. The high values indicated that the AgNPs are stable within the solution as the zeta potential value far exceeds the required minimum. The diameter sizes (Z-Average d. nm) observed from the DLS analysis, in Table 1, ranged from 30 to 53 d. nm, which were comparable to the particle size observed by the TEM results (Figure 3). The Polydispersity Index (PDI) values were 0.414, 0.442, and 0.48 for the leaf, stem and tuber biosynthesized AgNPs, respectively. These values demonstrated that the AgNPs were polydispersed.

**TABLE 1:** Dynamic light scattering and zeta potential assessment of the biosynthesized nanoparticles.

Synthesized AgNP	Zeta potential (mV)	Dynamic light scattering	
		Z-Average (d. nm)	PDI
Leaf AgNPs	−81.8 ± 0.9	41.87 ± 13.33	0.414 ± 0.042
Stem AgNPs	−88.5 ± 1.91	30.16 ± 6.98	0.442 ± 0.049
Tuber AgNPs	−83.5 ± 1.25	53.94 ± 19.37	0.48 ± 0.053

The zeta potential analyzer is a tool used to analyze the surface potential of NPs, i.e., the potential difference between the accessible surface of the NPs and the medium where the NPs are dispersed (Chandraker et al., 2021; Giri et al., 2022). This tool provides significant information on synthesized NPs. A zeta potential value of ± 30 mV is crucial of a stable nanosuspension (Aruna, 2014; Liaqat et al., 2022). Although general plant-mediated AgNPs exhibit zeta potentials of −20 to −40 mV, there have been instances of more negative values observed for varying plant species, as a result of the synthesis process, the ionizable phytochemicals adsorbing onto the AgNP surface or the dispersion medium used during the measurement. The high values are unusual but not unrecognized. Zeta potential of −80.7 mV had been observed in a study which synthesized AgNPs using the extracts of *E. spiralis* (Khalir et al., 2020). The negative value signified that the AgNPs are negatively charge causing an electrostatic repulsion between the AgNP which facilitates the stability of the AgNPs and prevent further agglomeration; it also may be due to the capping of NPs by the organic compounds from the plant (Edison and Sethuraman, 2012; Sivaraman et al., 2013; Liaqat et al., 2022).

PDI values that range from 0.001 to 0.1 indicate well dispersed AgNPs (highly monodispersed), 0.1 to 0.2 are considered moderately monodispersed, and values greater than 0.2 and higher indicates polydispersity which is due to aggregation and agglomeration which increase the particles size (Singh et al., 2014; Roseline et al., 2019; Jiang et al., 2023; NanoTemper Technologies, 2024). The size variation exhibited by the biosynthesized AgNPs could also be due to the presence of aggregated particles. These aggregated particles have a greater tendency to accumulate and form agglomerations. This phenomenon occurs due to factors such as the capping agent, synthesis conditions and surface ligands (Nanomicrospheres, 2024). One advantage of polydispersed NPs is that they are more enduring in applications that do not require perfect homogeneity such as in drug formulations where varying size particles can help better the distribution of particle throughout the body (Nanotechnology, 2024).

#### 3.1.4 Fourier transform infrared spectroscopy

**FIGURE 6:**
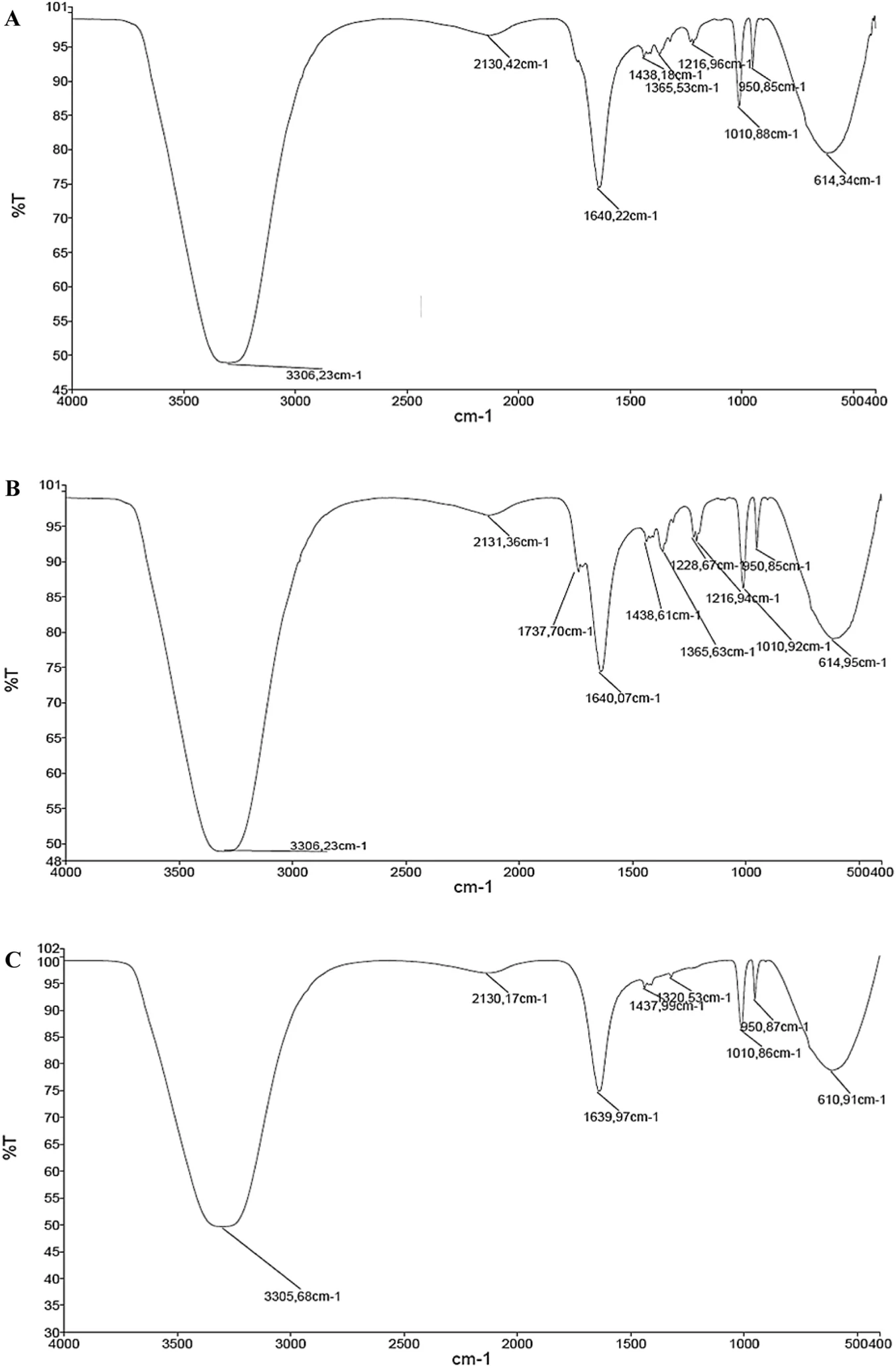
Fourier transform infrared spectroscopy of silver nanoparticles synthesized from *Kedrostis nana* leaf **(A)**, stem **(B)** and tuber **(C)** extract.

Figures 6A–C illustrate the functional groups of the AgNPs biosynthesized by *K. nana* leaves, stems and tubers using infrared spectroscopy. In Figure 6A, the leaf biosynthesized AgNPs showed absorbance bands at 3,306, 2130, 1,640, 1,438, 1,365, 1,216, 1,010, 950, and 614 cm^-1^. As observed in Figure 6B, the stem AgNPs illustrated bands at 3,306, 2131, 1737, 1,640, 1,438, 1,365, 1,228, 1,216, 1,010, 950, and 614 cm^-1^. The tuber biosynthesized AgNPs (Figure 6C) revealed bands at 3,305, 2130, 1,639, 1,437, 1,320, 1,010, 950, and 614 cm^-1^. The spectroscopy results showed similar bands for all biosynthesized AgNPs. Based on the works by Silverstein et al. (2014), the intense peaks at 3,305 cm^−1^ and 3,306 cm^−1^ indicate the presence of O-H stretching of phenolic compounds or N-H stretching vibrations, characteristic of amine or amide groups. The peak at 2130 cm^−1^ corresponds to C≡N stretching, suggesting the presence of nitrile groups.

A peak at 1737 cm^−1^ is associated with C=O stretching in saturated ester. Peaks observed between 1,639-1,640 cm^−1^ are attributed to C=C stretching in alkenes or aromatic rings. The 1,437-1,438 cm^−1^ region indicates C-H stretching of the methyl groups, while medium intensity peaks between 1,365-1,320 cm^−1^ correspond to C-H bending in alkyl groups and C–N stretching in amine groups. Peaks that are in the range of 1,228-1,216 cm^−1^ are associated with C-N stretching of amines. Absorption at 1,010 cm^−1^ and 950 cm^−1^ suggests C–C stretching in ethers or esters. And finally, a strong peak at 614 cm^−1^ represents out of plane C-H bending vibrations characteristic of aromatic carbons. The FTIR analysis represents a fingerprint of a sample represented by absorption peaks associated with the various functional groups could be attributed to, in this case, plant metabolites acting as the reducing and capping agent of the AgNPs (Jasmine et al., 2016; Salve et al., 2022).

### 3.2 Antioxidant assessment of the biosynthesized silver nanoparticles

**FIGURE 7:**
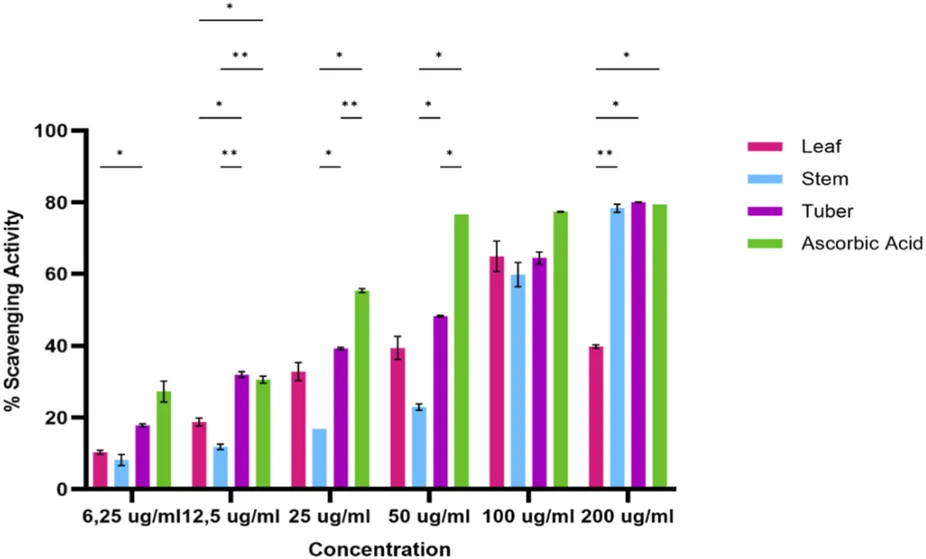
DPPH scavenging activity of *Kedrostis nana* biosynthesized silver nanoparticles.

Based on the results in Figure 7, the 200 μg/mL concentration of the stem and tuber biosynthesized AgNPs showed to have mean values of 78.39% and 80.08%, respectively, which were similar to the standard used, ascorbic acid (79.43%). The stem AgNPs, tuber AgNPs and the ascorbic acid standard were not statistically different. However, the biosynthesized AgNPs showed decreased scavenging activity at the lower concentrations (6.25 µg/mL- 100 μg/mL) compared to the ascorbic acid standard. Among the three NP preparations, the tuber-derived AgNPs showed greatest activity and showed radical scavenging activity comparable to the positive control. This superior activity may reflect the differences in phytochemicals present in the tuber which could influence NP synthesis and surface capping (Restrepo abd Villa, 2021). The presence of phenolic compounds confirmed by the FTIR analyses (Figures 6A–C) indicated that these compounds are attached to the AgNPs, thereby contributing to their radical scavenging ability. The antioxidant capacity of the synthesized AgNPs may be related to the medicinal plant’s phytochemicals, such as polyphenols, vitamins and carotenoids, coating the AgNPs (Urnukhsaikhan et al., 2021).

Similar observations have been reported for tuber and rhizome synthesized AgNPs from other medicinal plants. For example, *Dioscorea bulbifera* tuber extract was found to be rich in phenolics, flavonoids, reducing sugars and ascorbic acid. These compounds play important roles in the reduction and stabilization of AgNPs (Ghosh et al., 2012). Chaturvedi et al. (2020) showed that the AgNPs derived from the rhizomes of *Curcuma caesia* Roxb exhibited DPPH free radical scavenging activity from 20 μg/mL to 100 μg/mL.

The mechanism of the antioxidant activity of the AgNPs can also be explained by Ag existing in two oxidation states, namely, Ag^+^ and Ag^2+^, and depending on the condition of the reaction, the AgNPs may donate or accept electrons in order to quench free radicals (Shanmugasundaram et al., 2013; Bedlovičová et al., 2020). The higher antioxidant activity, as seen in the 200 μg/mL stem and tuber biosynthesized AgNPs, may be due to the interactions of the Ag ions with the functional groups of *K. nana* extracts which formed NPs that are spherical and contain a larger surface area, resulting in a high synergistic effect of the nanosized AgNP (Khane et al., 2022).

Understanding the antioxidant capacity of these synthesized AgNPs is important in human health as these may be able to inhibit or delay certain undesired oxidation reactions thereby helping to prevent oxidative stress usually linked to diseases such as neurodegenerative disorders, high blood pressure or cancer (Apak, 2019; Rumpf et al., 2023). The basis of the DPPH reaction is color fading when the radical is reduced by an oxidant (Salehi et al., 2016; Mukaratirwa-Muchanyereyi et al., 2022). This reduction is visible by the disappearance of its violet color and is associated with a decreased absorbance; thus, the antioxidant capacity determined is related to the removal of the radical (Martínez-cabanas et al., 2021). These results show that the AgNP have the potential to be used in drug delivery systems, anti-inflammatory applications and wound healing to reduce the oxidative damage at the site of the injury, cosmeceuticals such as UV protection and anti-aging formulations as the green synthesized AgNPs have displayed a similar stability to ascorbic acid (Urnukhsaikhan et al., 2021; Lakkim et al., 2003).

### 3.3 Antibacterial activity of the biosynthesized silver nanoparticles

**FIGURE 8:**
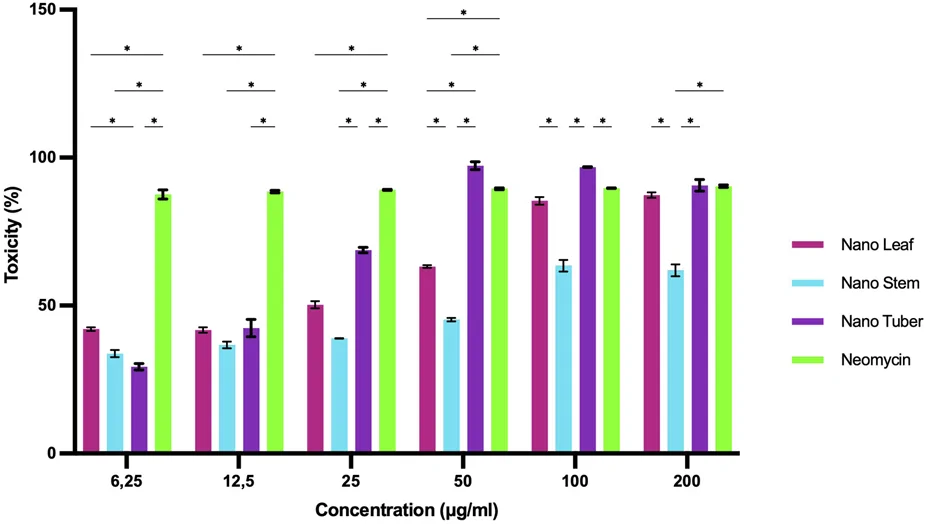
Antibacterial activity of *Kedrostis nana* biosynthesized silver nanoparticles against *Staphylococcus aureus*.

**FIGURE 9:**
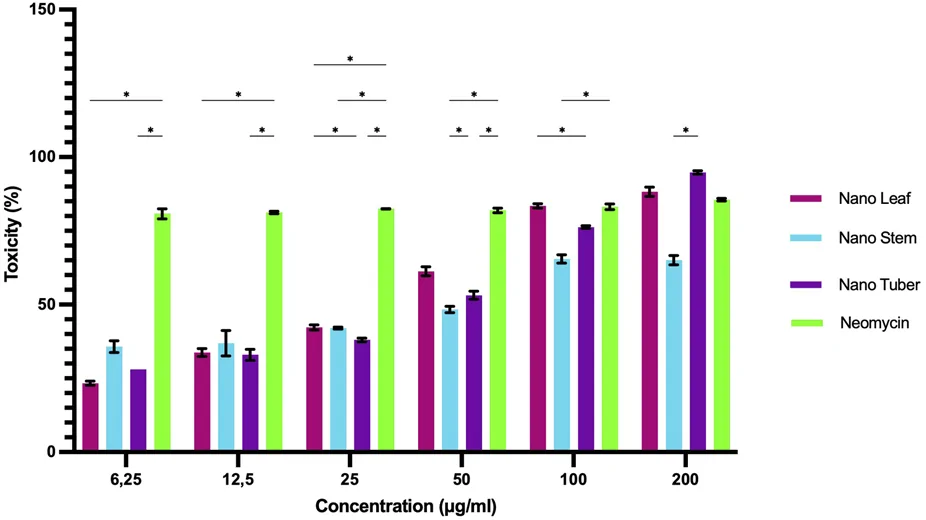
Antibacterial activity of *Kedrostis nana* biosynthesized silver nanoparticles against *E. faecalis*.

**FIGURE 10:**
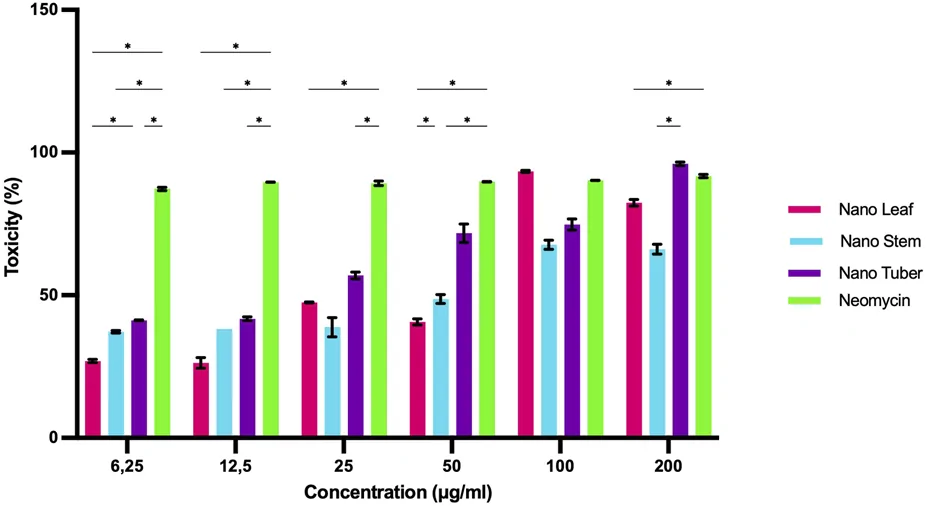
Antibacterial activity of *Kedrostis nana* biosynthesized silver nanoparticles against *P. aeruginosa*.

**FIGURE 11:**
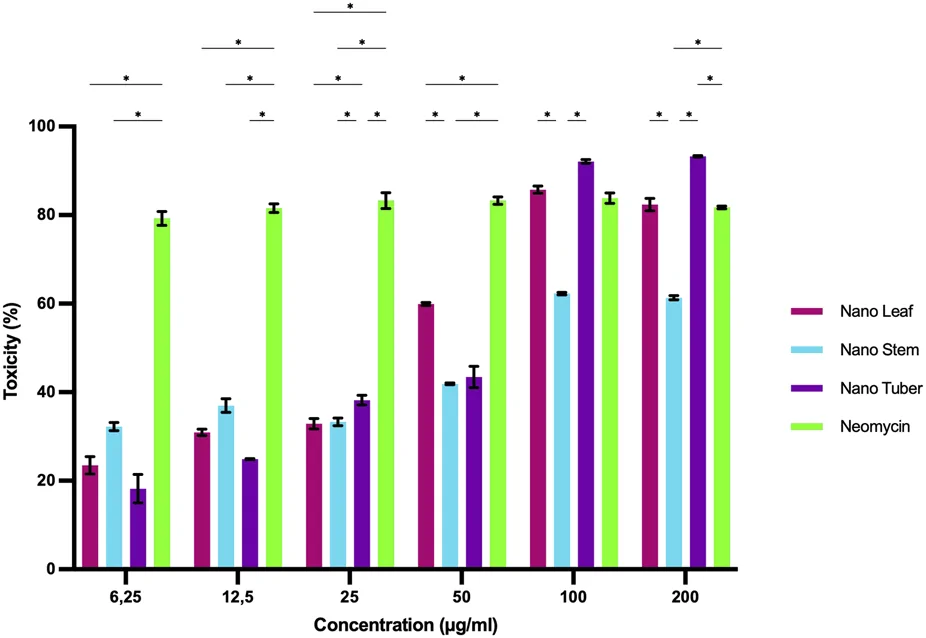
Antibacterial activity of *Kedrostis nana* biosynthesized silver nanoparticles against *E. coli.*

**FIGURE 12:**
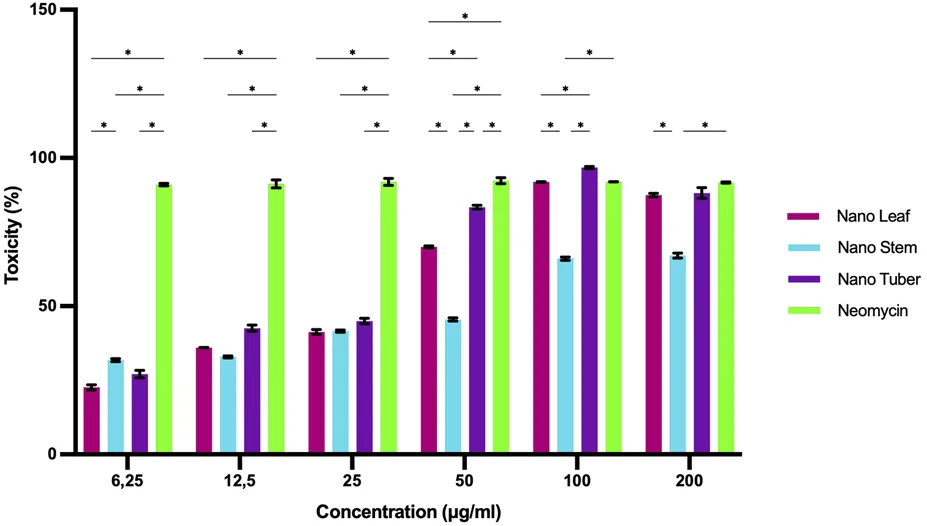
Antibacterial activity of *Kedrostis nana* biosynthesized silver nanoparticles against *K. pneumoniae*.

Based on Figures 8–12, the AgNPs displayed significant toxicity compared to the standard antibiotic neomycin. Figures 8, 9 illustrate the antibacterial activity of the biosynthesized AgNPs against the Gram-positive bacterial strains, *S. aureus* and *E. faecalis*. As seen in Figure 8, the percentage toxicity shown by the biosynthesized AgNPs against *S. aureus* was considerable. The lower concentrations did not perform well for all plant organs, however, at 50 μg/mL, the tuber biosynthesized AgNPs achieved a toxicity of 97.28%, outperforming all other concentrations, and was higher than the neomycin standard which exerted a toxicity of 89.47%. The tuber extract-derived AgNPs at 50 μg/mL was statistically different to the neomycin standard at the same concentration. At 100 μg/mL, the tuber AgNPs had a mean toxicity of 95.98% which was also higher than neomycin (89.67%). The tuber AgNPs and neomycin were statistically different from one another. At 200 μg/mL, the tuber AgNPs also had a higher percentage toxicity of 90.61% when compared to the standard which displayed a toxicity of 90.32%, however they were not significantly different.

Based on Figure 9, the tuber extract-derived AgNPs (94.82%) performed better than neomycin (85.55%) against *E. faecalis* at 200 μg/mL. These were statistically different to one another, however the leaf AgNPs at 100 μg/mL (83.46%) and 200 μg/mL (88.26%) also had higher toxicities compared to the standard (83.15% at 100 μg/mL). At both concentrations, the leaf AgNPs were not statistically different compared to the standard.

Figures 10–12 depict the antibacterial activity of the biosynthesized AgNPs against the Gram-negative bacterial strains, *P. aeruginosa, E. coli* and *K. pneumoniae*. According to the graph in Figure 10, it can be seen that the lower concentrations also did not perform well against *P. aeruginosa*. However, the percentage toxicity of the leaf AgNPs at 100 μg/mL had a mean toxicity value of 93.38% while the percentage toxicity of neomycin was 90.24%, and these were not statistically different from each other. At 200 μg/mL, the tuber AgNPs exhibited higher toxicity (96,06%) compared to the AgNPs synthesized from the other plant organs and neomycin (91.73%). The tuber AgNPs were statistically different to the AgNPs synthesized by the other plant organ NPs and neomycin. Figure 11 showed that at 100 μg/mL, the tuber extract-derived AgNPs (92.11%) had the highest percentage toxicity against *E. coli* compared to the standard which displayed 83.84% bacterial toxicity. At 200 μg/mL, the tuber AgNPs had higher percentage toxicity (93.27%) and was statistically different compared to ascorbic acid’s toxicity of 81.73%. Also noteworthy, the leaf extract-derived AgNPs performed significantly higher than the standard at 100 μg/mL (85.75%) and at 200 μg/mL (82.36%). Against *K. pneumoniae,* according to Figure 12, the tuber and the leaf extract-derived AgNPs at 100 μg/mL performed much better than all other concentrations, with toxicities of 96.78% and 91.88% when compared to the 91.95% toxicity of the standard. The toxicity exhibited by the tuber extract-derived AgNPs at 100 μg/mL was statistically different compared to the toxicity exhibited by neomycin.

The MIC for the bacterial strains is shown in Table 2. Against *S. aureus*, the MIC was determined at 100 μg/mL for the leaf and tuber AgNPs. The MIC was observed at 100 μg/mL for the leaf and stem AgNPs against *P. aeruginosa*. The leaf and tuber extract-derived AgNPs exhibited a MIC of 100 μg/mL against *E. coli*. The MIC of the stem and leaf extract-derived AgNPs was determined at 100 μg/mL against *E. faecalis*, while for *K. pneumoniae*, the MIC was ascertained for the leaf and tuber extract-derived AgNPs at 100 μg/mL.

**TABLE 2:** Minimum inhibitory concentration (µg/mL) of biosynthesized silver nanoparticles from *K. nana.*

Bacterial strain	AgNP-leaf	AgNP-stem	AgNP-tuber
*S. aureus*	100	100	50
*E. faecalis*	100	100	200
*P. aeruginosa*	100	100	200
*E. coli*	100	ND	100
*K. pneumoniae*	100	200	50

ND- Not determined. Complete inhibition of growth was not achieved and therefore MIC, values could not be ascertained.

Overall, the AgNPs synthesized from *K. nana* plant extracts showed excellent antibacterial activity. The leaf extract-derived AgNPs, had toxicities above 80% for all bacterial strains at concentrations 100 and 200 μg/mL. Particularly, against the Gram-positive strains, the AgNPs at 200 μg/mL had considerable antibacterial activity. While against the Gram-negative strains *P. aeruginosa* and *K. pneumoniae*, the AgNPs at 100 μg/mL performed exceptionally with toxicities over 90%. At the same concentrations, the stem extract-derived AgNPs did not perform as significantly as the leaf AgNPs against all bacterial strains, with toxicities only reaching between 60% and 70%. The highest toxicity was reached at 67% against Gram-negative *P. aeruginosa* at 100 μg/mL. Against the Gram-positive strains, the tuber extract-derived AgNPs had performed outstandingly with toxicities reaching above 90%. The highest toxicity observed at 50 μg/mL against *S. aureus* with a percentage toxicity of 97%. While against the Gram-negative strains, the highest toxicities were observed at 100 and 200 μg/mL against *K. pneumoniae, P. aeruginosa* and *E. coli*.

In a study by Kim et al. (2024), the antibacterial activity of AgNPs synthesized using mulberry leaves exhibited MIC values of 125, 250 and 500 μg/mL against *E. coli* and 125, 500 and 1000 μg/mL against *S. aureus*, therefore our leaf extract-derived AgNPs have demonstrated better activity with MIC values of 50 and 100 μg/mL for the same bacterial strains. In comparison to other studies on species within the Cucurbitaceae family, one study investigated the antibacterial activity of *Momordica charantia* AgNPs at concentrations of 1–64 μg/mL. The AgNPs synthesized exhibited MIC values of 4, 2 and 4 μg/mL against S*. aureus, P. aeruginosa* and *E. coli, r*espectively (Moorthy et al., 2021). According to a study by Aktepe and Baran (2022), the MIC values obtained from the antibacterial assay using AgNPs derived from *Cucurbita maxima* fruit were 0.12, 2, and 1 μg/mL against S*. aureus, P. aeruginosa* and *E. coli, r*espectively. The bitter apple seed was investigated in another study for its antibacterial activity (El-Beltagi et al., 2026). The seeds were used to synthesize AgNPs and the INT assay was used to evaluate the NPs antibacterial potential. The MIC values exhibited by these NPs were 12 and 25 μg/mL against *S. aureus* and *P. aeruginosa*, respectively. Antibacterial activity evaluation via the INT assays using AgNPs synthesized from Cucurbitaceae species are extremely limited. This makes it difficult to execute comparative assessments within this family.

According to the findings in this assay, the tuber AgNPs proved to be a viable candidate as an antibacterial agent against the various Gram-positive (*S. aureus* and *E. faecalis*) and Gram-negative (*P. aeruginosa*, *E. coli* and *K. pneumoniae*) bacterial strains. There are numerous studies that demonstrated the AgNP’s effectiveness against Gram-positive and Gram-negative bacterial infections (Shirley et al., 2010; Bolatchiev, 2020; Arya et al., 2024). The FTIR results (Figures 6A–C), show the presence of phenolic compounds that have originated from the plant extracts which are attached to the surface of the AgNP where they act as a capping and stabilizing agent on the surface of the NPs. These phenolic compounds enhance the antibacterial efficacy of the NPs by improving bacterial membrane interactions (Xu et al., 2025). It can be assumed that these compounds enhanced the antibacterial potential of the AgNPs synthesized.

Other studies have shown that AgNPs synthesized from the tuber or bulbs also have potent antibacterial activity. One study showed that *Dioscorea bulbifera* had exhibited a rich diversity of phytochemicals such as phenolics, flavonoids, carbohydrates and vitamins. These compounds are speculated to have played a significant role in the bio reduction of AgNPs. The study also showed antibacterial activity against *S. aureus*, *P. aeruginosa*, *E. coli* and *K. pneumoniae* with the highest zone of inhibition observed at 1,000 μg/disc (Ghosh et al., 2012). *Allium cepa* bulb-derived AgNPs have also shown considerable antibacterial activity against *S. aureus*, *E. coli*, *K. pneumoniae* and *P. aeruginosa* (Gomaa, 2017). The bulb-derived AgNPs displayed MIC values of 0.312 mg/mL for *S. aureus* and *E. coli,* 2.5 mg/mL for *K. pneumoniae* and 0.156 mg/mL for *P. aeruginosa*. Similarly, Mosaviniya et al. (2019) exhibited that the AgNPs synthesized from the bulbs of *Crocis haussknechtii* bois had strong inhibitory effect against *S. aureus* and *P. aeruginosa* with MIC values of 26.9 and 20.2 μg/mL, respectively. Pugazhendhi et al. (2016) synthesized AgNPs from the tubers of *Dioscorea alata.* They confirmed the antibacterial activity of the AgNPs using the agar well diffusion assay method against *E. coli* and *S. aureus*.

Based on literature on bacterial strains, Gram-positive bacteria have a thick peptidoglycan layer in their cell wall which provides structural integrity and influences susceptibility to certain antibiotics (Only Zoology, 2024). The Gram-negative bacterial strains possess a thinner peptidoglycan layer which is located between two membranes, thus making the antibiotic treatment complicated as this outer membrane acts as a barrier (Only Zoology, 2024). AgNPs are, however, able to interact with the cell wall of bacteria, disrupting the cells’ integrity and causing the phosphodiester linkages to break down leading to the death of the bacterium. Additionally, the Ag ions can bind to various crucial biological components within the cell such as nitrogen, oxygen and sulphur, which impedes the growth of the cell (Juan et al., 2010; Arya et al., 2024). Because of the green method of synthesis used, the phytochemicals on the surface of the AgNPs enhance their bioactivity (Wypij et al., 2021).

The biological based synthesis of NPs is preferred over conventional chemical and physical techniques as these are costly, more labor intensive and includes the use of hazardous chemicals, while the green synthesis method uses biological templates that provide an ecofriendly alternative that is sustainable, less toxic and enhanced biocompatibility (Chandraker et al., 2020; Elsaffany et al., 2025). However, the overall antibacterial activity of the AgNPs could be influenced by variations in the synthesis method, stabilizing agents and the bacterial resistance mechanisms (Franco et al., 2022). To combat these bacterial resistance mechanisms, various conventional strategies could be implemented. These include microbial-based therapies, immunotherapeutic modalities, stem cell applications, photodynamic antimicrobial techniques, bacteriophage therapy, quorum quenching mechanisms that interfere with communication networks in bacteria, bioactive compounds from animal venom and CRISPR-Cas system with its complementary resistance mechanisms (Butler et al., 2023; Elshobary et al., 2025; Al-Harbi et al., 2025). NPs are superior in that there is physical damage to the bacteria, for example, the cell walls of the bacteria may become damaged due to the absorption and penetration of NPs, they interact directly with the bacteria’s genetic material and enzymes which lead to protein inactivation or replication inhibition due to ion diffusion; they are also multitargeted which makes it more difficult for the bacteria to adapt (Butler et al., 2023; Mondal et al., 2024; Elshobary et al., 2025; Al-Harbi et al., 2025).

### 3.4 Cytotoxicity assessment

The HEK293 cells serve as non-cancerous human cells for assessing biocompatibility and general cytotoxicity of the AgNPs, whereas the HeLa cell lines are well-established human cervical cancer cells that are commonly used to investigate the anticancer activity of the AgNPs. The use of both cell lines facilitates in comparing the effect of the AgNPs on non-malignant and malignant cells.

**FIGURE 13:**
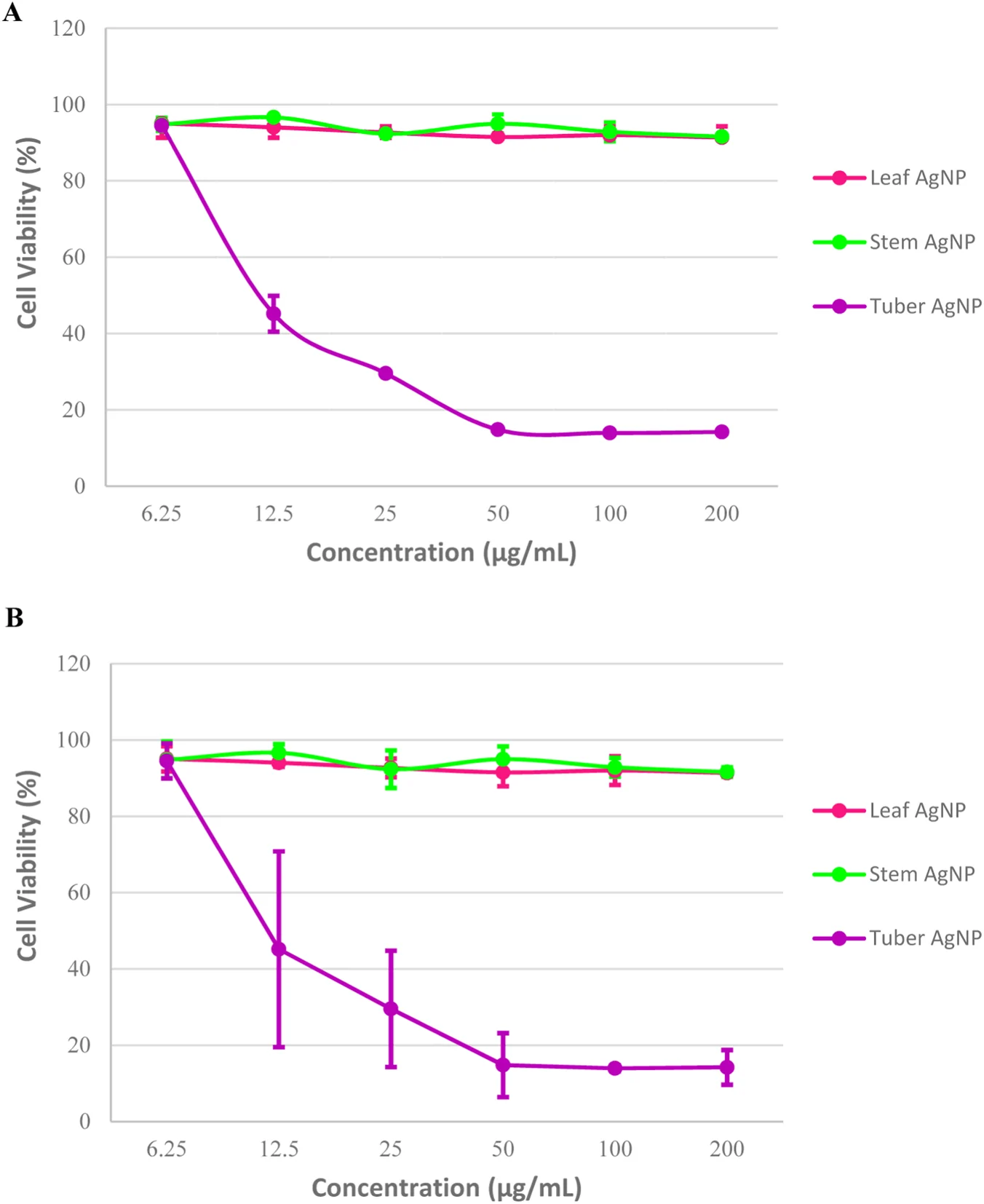
Effect of *K. nana* biosynthesized silver nanoparticles on Hek293 **(A)** and HeLa **(B)** cell viability.

Based on Table 3 along with Figures 13A,B, it was observed that the leaf and stem-derived AgNPs showed minimal toxicity to the non-cancerous human embryonic kidney (Hek293) cells as the percentage cell survival was relatively high with cell survival rates above 90% at all concentrations. In contrast, the tuber extract-derived AgNPs exhibited toxicity at higher concentrations. In Figure 13A, it can be observed that there is a sharp decline in cell viability from 94.5% at 6.25 μg/mL to 45% at 12.5 μg/mL. This trend continued to decrease to 15% at 50 μg/mL at 100 and 200 μg/mL, the cell viability remained moderately constant. It must be noted that only a concentration of 17.6 μg/mL is required to kill 50% of the cells.

**TABLE 3:** Cell survival rate (%) of Hek293 and HeLa cell lines after exposure to silver nanoparticles derived from the leaf, stem and tuber extracts of *K. nana*.

Concentration (µg/mL)	Hek293 (%)			HeLa (%)		
	AgNP-leaf	AgNP-stem	AgNP-tuber	AgNP-leaf	AgNP-stem	AgNP-tuber
6.25	95.08 ± 0.12	94.81 ± 1.78	94.53 ± 0.82	80.83 ± 3.32	98.79 ± 4.80	89.90 ± 2.50
12.5	94.06 ± 0.86	96.66 ± 0.21	45.17 ± 4.69	76.42 ± 1.05	86.15 ± 2.27	82.16 ± 0.72
25	92.70 ± 1.61	92.37 ± 0.11	29.54 ± 0.60	74.02 ± 2.44	79.78 ± 4.93	48.99 ± 3.67
50	91.53 ± 0.94	94.99 ± 2.45	14.81 ± 0.39	73.82 ± 3.64	80.36 ± 3.34	31.39 ± 1.89
100	92.01 ± 0.05	92.87 ± 2.51	13.96 ± 0.23	72.88 ± 3.76	75.04 ± 2.45	20.56 ± 0.45
200	91.38 ± 0.58	91.61 ± 0.48	14.21 ± 0.34	72.02 ± 0.43	73.98 ± 1.22	19 ± 0.90
IC_50_ (µg/mL)	>200	>200	17.6	>200	>200	33.49

All biosynthesized AgNPs showed toxicity against the human cervical carcinoma (HeLa) cell lines. The leaf and stem extract-derived AgNPs had no significant effect on the cell survival rate of the cancerous cells (Figure 13B). In comparison, the tuber derived AgNPs exhibited increased cytotoxicity to the HEK293 cells compared to the HeLa cells at lower concentrations. The tuber extract-derived AgNPs displayed significant cytotoxic potential against the cancerous cell lines as concentrations increased with an IC_50_ value of 33.49%. At 200 μg/mL, the tuber AgNPs had significant toxicity against the cancer cell lines with 19,76% cell survival rate. As the concentrations decreased, there was an increase in HeLa cell viability. The results in Table 3 indicate that the tuber-derived AgNPs did not exhibit selective cytotoxicity. The differences in the AgNPs cellular response may be due to variations in NP uptake, composition of the membrane and intracellular processing between the cancerous and non-cancerous cell lines (Hemlata et al., 2020; Kovács et al., 2022). Although the tuber-derived AgNPs showed cytotoxic activity, the greater sensitivity of the HEK293 cell line does suggest limited tumour selectivity. The extremely high IC_50_ values observed for the leaf and stem-derived AgNPs reflect the minimal cytotoxicity exhibited over the concentration range as the cell viability remained above 90%. As such, the linear regression model extrapolated beyond the experimental data to estimate the IC_50_ concentrations. These values indicate that the IC_50_ exceeded the highest concentration tested, rather than representing experimentally determined IC_50_ values (supplementary material).

Cytotoxic efficacy is linked to NP size and stability. In general, smaller NPs show increased cytotoxic efficacy because of the larger surface area and improved interaction with cellular components (Khan et al., 2025). Metal NPs at high concentrations have been reported to interfere with colometric assays such as MTT assays by means of adsorption of dyes, light scattering or interactions with formazan (Díaz et al., 2008; Mello et al., 2020; Nath et al., 2024). The decrease in viability was observed to be concentration dependent for the tuber derived-AgNPs, which suggests that the cytotoxicity was driven by biological effects. The phytochemicals present in the tuber extract-derived AgNPs, such as phenolic compounds exhibited in the FTIR analyses (Figures 6A–C), help to stabilize the NPs which can help to increase delivery into cancer cells. It is a necessity to perform cytotoxicity tests to screen chemicals for their toxic potential, especially on normal cell lines, as this can have harmful effects on human health (Castaño and Gómez-Lechón, 2005; Anywar et al., 2022).

Cancer is one of the leading causes of morbidity and mortality globally. It accounts for millions of new cases and deaths each year. Many countries suffer from inadequate access to cancer prevention protocols, screening, diagnostic services and quality cancer treatment, which leads to late diagnosis and unfortunate health outcomes (World Health Organization, 2020; Gaobotse et al., 2023). Globally, cervical cancer is the most commonly diagnosed cancers among women (Sung et al., 2021; WHO, 2024). It represents a significant health concern and the need for continued development of novel therapeutic agents is essential. In South African women, the most diagnosed type of cancer includes cervical, breast, colorectal and squamous cell carcinoma, while in men, the most common include prostate, lung, squamous cell and basal cell carcinoma (Finestone and Wishnia, 2022). Finding an alternative treatment source is therefore crucial. In correlation to the plants’ ethnomedicinal use as an anticancer agent, this study shows that the AgNPs biosynthesized from *K. nana* does have the potential to terminate cancer cells, however, more research is required due to the toxicity to non-cancerous cells.

## 4 Final conclusion

This study revealed that biosynthesized AgNPs had an average size range of 28, 21 and 26 nm for the leaf, stem and tuber AgNPs, respectively. These AgNPs were stable, monodispersed and negatively charged due to the capping of the AgNPs by plant phytochemicals. The stem and tuber AgNPs showed to have significant antioxidant potential at 200 μg/mL concentration; while against the five bacterial strains, the leaf and tuber AgNPs showed substantial antibacterial activity compared to the known antibiotic. However, the AgNPs exhibited cytotoxicity to the normal cell line, Hek293, at the concentration required to inhibit bacterial growth. The cytotoxicity assessment exhibited results that highlight the possibility of the tuber AgNPs being used as an anticancer agent as the AgNPs exhibited an IC_50_ of 33.4 μg/mL. Overall, the tuber extract-derived AgNPs exhibited significant biological activity compared to the leaf and stem extract-derived AgNPs. These results correlate to the plants’ use in traditional medicine.

The findings of this study demonstrated that *K. nana* is an appropriate reducing and stabilizing agent for the green synthesis of AgNPs. Unlike previous studies that have focused on single plant organs or other Cucurbitaceae species, this study presents a comparative assessment of AgNPs synthesized from different plant organs and their associated bioactivity. These findings contribute to the expanding knowledge on plant-mediated NP synthesis and also identifies *K. nana* as a promising South African medicinal plant for nanobiotechnological applications.

Certain aspects were beyond the scope of the initial investigation. These include the assessment of the extract stability and compositional consistency in the synthesis process. A separate DMSO vehicle control was not included. Although DMSO was used as the solvent for the AgNP stock solution, future studies should include a vehicle control to confirm that the solvent did not contribute to the observed bioactivity. Additionally, the long-term stability of the AgNPs as well as the release of silver ion under physiological conditions were not investigated. These factors could account for the observed cytotoxicity toward the Hek293 cell lines. These limitations do not detract from the antibacterial findings but rather introduce opportunities for future recommendations.

In order to decrease the cytotoxicity of the AgNPs and still retain their antibacterial activity, investigations into the improving phytochemical capping and surface stability as well as refining synthesis conditions to control particle size need to be conducted. Additionally, assessing the long-term stability as well as clinical applicability is necessary. *In vivo* safety and efficacy and apoptosis related assays, such as caspase activity or Annexin V/Pi staining, are essential to further characterize the biological effects of the synthesized AgNPs. Synthesizing NPs from plant extracts has benefits over other biological systems such as microorganisms, i.e., it is a simpler method to handle, there is less biohazard waste, and no inflated costs required to maintain microbial isolations and culture mediums. This study is the first to report on the biosynthesis of AgNPs from *K. nana* extracts as well as their characterization and biological activity.

## Data Availability

The data presented in the study are deposited in the Yabelana repository, accession number ba2fd08dc00ac11e2fac and can be accessed using the URL below: https://figshare.com/s/ba2fd08dc00ac11e2fac.
